# ‘Workable utopias’ for social change through inclusion and empowerment? Community supported agriculture (CSA) in Wales as social innovation

**DOI:** 10.1007/s10460-020-10141-6

**Published:** 2020-08-18

**Authors:** Tezcan Mert-Cakal, Mara Miele

**Affiliations:** 1 Cardiff University Alumna, School of Geography and Planning, Glamorgan Building, King Edward VII Avenue, Cardiff, CF10 3WA Wales UK; 2 School of Geography and Planning, Cardiff University, Glamorgan Building, King Edward VII Avenue, Cardiff, CF10 3WA Wales UK

**Keywords:** Community supported agriculture, CSA, Social innovation, Alternative food, Grassroots initiatives, Food sustainability

## Abstract

The focus of this article is community supported agriculture (CSA) as an alternative food movement and a bottom-up response to the problems of the dominant food systems. By utilizing social innovation approach that explores the relationship between causes for human needs and emergence of socially innovative food initiatives, the article examines how the CSA projects emerge and why, what is their innovative role as part of the social economy and what is their transformative potential. Based on qualitative data from four different models of CSA case studies in different regions of Wales, UK, and by using concepts from an alternative model for social innovation (ALMOLIN) as analytical tool, the article demonstrates that the Welsh CSA cases play distinctive roles as part of the social economy. They satisfy the needs for ecologically sound and ethically produced food, grown within communities of like-minded people and they empower individuals and communities at micro level, while at the same time experiment with how to be economically sustainable and resilient on a small scale. The paper argues that in order to become ‘workable utopias’, the CSA initiatives need to overcome the barriers that prevent them from replicating, participating in policies and decision-making at macro level, and scaling up.

## Introduction

The growth in alternative food networks (AFNs) in late 1980s and early 2000s is indicative of the bottom-up responses to the unsustainable food systems that are increasingly unable to address the needs and demands of food producers and consumers alike (Sage 2014). Farmers' markets, box schemes, community supported agriculture (CSA), producer and consumer co-operatives, and community gardening initiatives are all examples of such AFNs (Jarosz 2008). Bos and Owen (2016) argue that these types of food provisioning systems are significantly different to conventional counterparts as they can redefine relations between producers and consumers through transparent short(er) food supply chains. These are founded upon quality and provenance and point towards more sustainable modes of production (Marsden et al. 2000; Renting et al. 2003; Sage 2003; Goodman 2004; Ilbery and Maye 2005; Morris and Kirwan 2011).

The recent COVID-19 pandemic exposed not only the vulnerabilities and risks of the current food systems, specifically of the longer supply chains, but also its deep inequalities and injustices (Anderson 2020). On the one hand, many places around the world have faced empty supermarket shelves while on the other, crops were left to rot on the field due to restriction of movement, causing shortage of seasonal workers (Hendrickson 2020; Gustin 2020). These were further exacerbated by disruption in logistics and shortage of animal feed and fertilizers (Roy Chaudury 2020; Fertilizers_Europe n.d.). On international level, since most of the grains are traded across borders as a result of the trade liberalization of 1980s, there have been fears of shortage of staples as some of the exporting countries restricted the supply, which reminded of 2007–2008 food crisis (IPES-Food 2020). However, as much as the COVID-19 pandemic exposed how fragile and unsustainable the global food systems are in the event of shock, it also demonstrated the resilience of the local food initiatives and short food supply chains. CSA schemes in many countries saw increased customer numbers while the interest to local box schemes grew dramatically (Schmidt et al. 2020; URGENCI 2020). For example, a survey of the vegetable box schemes in the UK revealed an overall increase in sales by 111% during the pandemic, with increase in small boxes by 134%; 10% of these schemes created systems to help those who were economically vulnerable (Wheeler 2020). Furthermore, local initiatives created platforms to collaborate with each other, exchange produce and help people in need. For instance, The CSA UK Network published online resources to help the initiatives cope with the COVID-19 conditions and increased demand (CSA 2020).

In this paper, we examine the role and transformative potential of the CSA in Wales (UK) by utilizing an alternative social innovation approach that explores the relationship between causes for human needs and emergence of socially innovative food initiatives (González et al. 2010). The CSA is an innovative idea bringing consumers and producers together, where consumers share the risks and benefits of production (Hinrichs 2000; Hayden and Buck 2012) and both sides mutually resolve some uncertainties (Lamine 2005). It started in 1970s in Japan and Switzerland and spread later to other parts of the world. The International CSA Network URGENCI was launched in 2008, and the CSA Network UK followed in 2013. The existing literature does not offer much evidence about how these initiatives emerge, what resources are mobilized and what needs they aim to address. Secondly, little research has focused on how the CSA empowers individuals and communities. And finally, the existing studies examining the CSA from social innovation perspective tend to favour socio-technical transition frameworks based on niche-regime interaction (Brunori et al. 2010; Marsden 2013; Rossi 2017), where niches are spaces for experimenting with innovative ideas and regime is the complex of settled institutions, policies, regulations, actors and relations (Kemp et al. 1998; Smith 2007; Schot and Geels 2008). Therefore, our threefold aim is to address these gaps in the literature: first, by examining how the CSA initiatives in Wales emerge and why; second, by exploring their innovative role as part of the social economy; and third, by scrutinizing their transformative potential through an alternative social innovation framework. We use data from four CSA schemes in Wales as qualitative case studies based on participant observations and semi-structured interviews. The paper proceeds in the following way: first we review the literature about social innovation, CSA and AFNs; secondly, we explain our methodological approach, then we present the four cases in Wales and the results that correspond to the research aims; and finally, we discuss their theoretical and practical implications before concluding with areas for further research.

## Social innovation, CSA and AFNs

Social innovation is defined by Gilles Deleuze as “opportunity spaces at micro scales [that] may make creative strategies possible at macro scales” and as a way of “building ‘workable utopias’”; it is about countering the conservative forces eager to preserve social exclusion situations, thus being “an ethical position of social justice” (Moulaert et al. 2013, p. 17). A source for social innovation is the social economy (Howaldt et al. 2014), which is an alternative vision for the economy, different from its neoliberal representation as ‘a monolithic entity’ detached from social life (Jessop et al. 2013). MacCallum et al. (2009, pp. 1–2) explain that social innovation emerges through collective action, social movements or public policy to address social problems like exclusion, deprivation and lack of wellbeing, and improve human conditions through satisfaction of needs, empowerment and improvement of social relations, also defined as the three main dimensions of social innovation (Moulaert et al. 2005), namely (1) product dimension, which focuses on the satisfaction of ‘alienated’ needs that have not yet been satisfied or are no longer considered as important by mainstream actors, (2) empowerment dimension, about increasing the socio-political capabilities and access to resources required to satisfy those needs, and (3) process dimension, about the change in social and governance relations.

Regarding the product dimension, firstly, identifying the needs is essential to understand what triggers the emergence of social innovations. According to Parra (2013), needs in social innovation can be material and existential and may be collectively defined by communities. Therefore, we ask why people participate in the CSA schemes. The closest answer that the existing literature offers is about motives for participation rather than needs. For the consumers, food safety concerns and knowing the source of their food (Cooley and Lass 1998; Goland 2002), acquiring quality and nutritious produce (Sharp et al. 2002; Farmer et al. 2014), addressing environmental concerns, and supporting local farmers (Goland 2002; MacMillan Uribe et al. 2012; Farmer et al. 2014) are primary motives while for the producers, these range from providing organic and seasonal produce for local people (Cox et al. 2008) to accessing larger markets, increasing awareness of the food systems, and building stronger community (Sharp et al. 2002). But although motives demonstrate why people participate to the schemes, there is lack of evidence in the literature about the needs that create deprivation and exclusionary circumstances in the case of the CSA. Furthermore, identifying the needs will enable us to establish if the product dimension of social innovation has been addressed. Again, despite an abundance of research on the various benefits of the CSA, such as health benefits (Ostrom 2007; Cohen et al. 2012; Minaker et al. 2014; Wilkins et al. 2015; Wharton et al. 2015; Allen IV et al. 2017), lifestyle changes (Ostrom 2007), impact on the participants’ environmental ethics (Hayden and Buck 2012), higher benefits for lower-income members (Galt et al. 2016), meeting psychological needs (Zepeda et al. 2013), and being powerful approach to food justice (Gottlieb and Joshi 2010, p. 149), the existing studies do not provide much evidence about the role of the CSA initiatives in satisfying the unmet needs causing their emergence.

The empowerment dimension of social innovation is about increasing the socio-political capabilities of individuals and communities (Moulaert et al. 2005) by including people in decision-making and service provision and creating common visions for change (González et al. 2010). Empowering people means increasing the recognition, access and voice rights of marginalised groups (Martinelli 2010). Renting et al. (2012) suggest that access to healthy food in a socially inclusive way and engagement in food growing is a way of empowerment. In addition, building strong community is a way of increasing their socio-political capabilities of the CSA initiatives, also considered “a major selling point” in attracting more members (Schnell 2007, p. 559). There is a positive correlation between community capital and the retention of members (Flora and Bregendahl 2012). Many studies report difficulties about building strong community in the CSA, mainly related to attracting and retaining members, attributed to disappointment of the type and amount of produce (Hinrichs 2000; Ostrom 2007; Janssen 2010; Hayden and Buck 2012), or little interest among the members in participating to community events. Consequently, maintaining the community side is left to “already overworked CSA farmers” (Hinrichs 2000, p. 300), and finding and retaining members in many cases happens at the expense of the producers’ self-exploitation (Galt 2013). Studies suggest that targeting people who are committed to environmental values (Goland 2002) and better communication between producers and consumers are ways of increasing consumers’ commitment in longer term (Cox et al. 2008). However, as much as the existing research examines the issues related to social capital, it does not offer much evidence about other ways of empowerment in the CSA, such as learning or participating in decision-making, which can enhance the capabilities and the voice rights of people.

The process dimension of social innovation is not only about changing relations between individuals, but at macro-level it is about changing the governance relations between market economy and social economy and reorganising the power dynamics between the state, civil society and the market (González et al. 2010). It is related to the transformative potential of the CSA and AFNs in general. On the positive side, these networks are seen as a response to the growing problems of the conventional food system (Mount et al. 2013), as innovative means of the social economy juxtaposed to the market economy, named ‘seeds of change’ (Seyfang 2009, p. 74), as ‘diverse economies’ (Gibson-Graham 2008, p. 2), or as ‘the new moral economy’ based on ethical values in contrast to the neo-liberal economy (Morgan et al. 2006, pp. 166–167). Moreover, AFNs are believed to possess the ability to “reconvene trust between food producers and consumers” and “articulate new forms of political association and market governance” (Whatmore et al. 2003, p. 389). One of the criticisms that AFNs face is their inability to tackle social injustices by serving predominantly middle-class consumers (Renting et al. 2012) and white people (Guthman 2008a) and perpetrating social inequalities instead of including disadvantaged populations (Matacena 2016), defined as “narrow ‘class diet’ of privileged income groups” (Goodman 2004, p. 13). It is suggested that the obscure inequalities and injustices created by the AFNs are caused by their focusing exclusively on local values, named ‘defensive localism’ (Hinrichs 2003; Winter 2003) or ‘un-reflexive’ localism (DuPuis and Goodman 2005). Particularly the CSA schemes are criticized for serving mainly those who have the necessary education, income and time to commit (Cone and Myhre 2000) or affluent consumers (Selfa and Qazi 2005) by being predominantly located in areas populated by middle and upper middle class (Schnell 2007), and for creating “marginalization and powerlessness” by excluding certain groups (Farmer et al. 2014, p. 323, emphasis original). A counter-argument states that both ‘social inclusion’ and ‘social exclusion’ are contested terms, as not participating can also mean a choice (Shortall 2008). And although some studies suggest that the CSA schemes can become more inclusive by being sensitive to ethnicity and class positions (Caraher and Dowler 2014; Galt et al. 2016), the existing literature does not offer concrete examples of how these initiatives address the social injustice and exclusion.

Another criticism regarding the transformative potential of the AFNs is about their failure to counter the corporate food regime and transform the food systems. Food movements are accused of trying to solve social problems by placing responsibilities on individuals, which results only in changes at market level rather than state level, or changes in local policies rather than national policies (Guthman 2008b; Alkon and Mares 2012; Fairbairn 2012). Therefore, regarding the position of alternative food initiatives against the dominant food system, Watts et al. (2005) distinguish between ‘weak’ alternatives, that put emphasis on ‘local’ and can be subordinated by the conventional food supply chains, and ‘strong’ alternatives that can provide spatial, social and economic alternatives to conventional networks. Follett (2009) suggests that ‘weak’ alternatives adopt customs of both alternative and conventional networks, while ‘strong’ alternatives are guided by the customs of moral economy, such as human and animal welfare, community-building, supporting small scale farmers, ecological sustainability, trust and transparency. When it comes to the ways in which the alternative food movement can scale up, Wiskerke (2009) offers a holistic approach by bringing the concept of alternative food geographies, which combine public procurement, urban food strategies and AFNs. In a similar way, Matacena (2016) states that urban food policies can provide outlets and growing spaces for the AFNs through infrastructure, spatial planning and public procurement. And finally, Blay-Palmer et al. (2016, p. 39) suggest building a “System of Sustainable Food Systems (SoSFS) as a counter-point to the corporate food regime” and based on community food networks connected via sharing good practices and knowledge. However, the literature does not provide much insight about how the actors of the CSA initiatives view their position and values against the main food system. Yet another gap is the lack of comparative evaluation of the barriers and opportunities for scaling up different CSA models.

In sum, the review of the literature on AFNs and CSA revealed several gaps related to each dimension of social innovation. Regarding the product dimension, there is a lack of studies about the needs that trigger the emergence of the CSA initiatives and the way these needs are satisfied. Regarding the empowerment dimension, apart from building strong community, there is not much evidence about learning and decision-making as other processes of empowerment. And the process dimension is under-researched in terms of the actors’ perspective on the position and values of the CSA against the main food economy, the ways of addressing the social injustice and exclusion, and comparative evaluation of the barriers and opportunities for transformation of different CSA types. We address these gaps by searching an answer to the main question of this study: how the CSA initiatives in Wales can become ‘workable utopias’ for food systems’ change through social inclusion and empowerment. More specifically, we ask (1) what are the needs that cause exclusionary circumstances and lead to the emergence of the CSA initiatives in Wales and how are these needs satisfied; (2) how do learning and participating in decision-making at various levels empower people; (3) what are the actors’ perceptions about the position and values of the CSA against the main food system; (4) how do CSA farms and gardens tackle social injustice; and (5) what are the barriers and possibilities for transformation of different CSA types in Wales that create ‘path-dependency’.^1^

## Methodology

To carry out this research, we used qualitative case studies based on semi-structured interviews and participant observation in four CSA initiatives in Wales. Case studies are particularly suitable as a method in understanding ‘how’ and ‘why’ the CSA initiatives operate (Yin 2003) and the ‘real-life’ phenomena unfold in practice (Flyvbjerg 2006, p. 235). Moreover, they are well suited to understand the systems, everyday practices, and the relations between the actors of the CSA initiative, that requires a “qualitative, context-sensitive [and] interactive” type of approach (Hamdouch 2013, p. 260). And lastly, case studies allow some general conclusions to be drawn through ‘theoretical reasoning’ (May and Perry 2011, p. 223). The four cases were selected based on two criteria: different locations and different ownership models. We used two sources to identify the potential cases. The first was the Federation of City Farms and Community Gardens (the Federation), which is the umbrella organisation of all community food growing projects in the UK (now renamed Social Farms and Gardens), and the second was the Soil Association,^2^ which at the time listed on its website the Welsh CSA projects, later transferred to the website of the newly-launched CSA UK Network.^3^

The data was collected between July 2014 and February 2015 with 3–5 days spent in each CSA by being actively involved as volunteer in the daily works of the initiatives and doing semi-structured interviews and observations. Interview was chosen as method due to its usefulness in explaining the complexity of processes and systems in detail and to investigate personal approaches and perceptions (Ritchie 2003; Cloke et al. 2004, pp. 150–151). Participant observation of daily activities at the CSA sites allowed us to gain additional insights by having direct access to the phenomenon (Laurier 2010) and provided a more objective perspective since interviews can sometimes be biased (Hancock and Algozzine 2006, p. 46) and cannot be replicated (Valentine 2005). We conducted 19 interviews in total with various actors involved in these initiatives, e.g. growers, members and volunteers and combined these with the observation notes during the analysis. We also revised related policy documents, reports, and media articles to establish a general picture of the CSA in Wales.

**Fig. 1:**
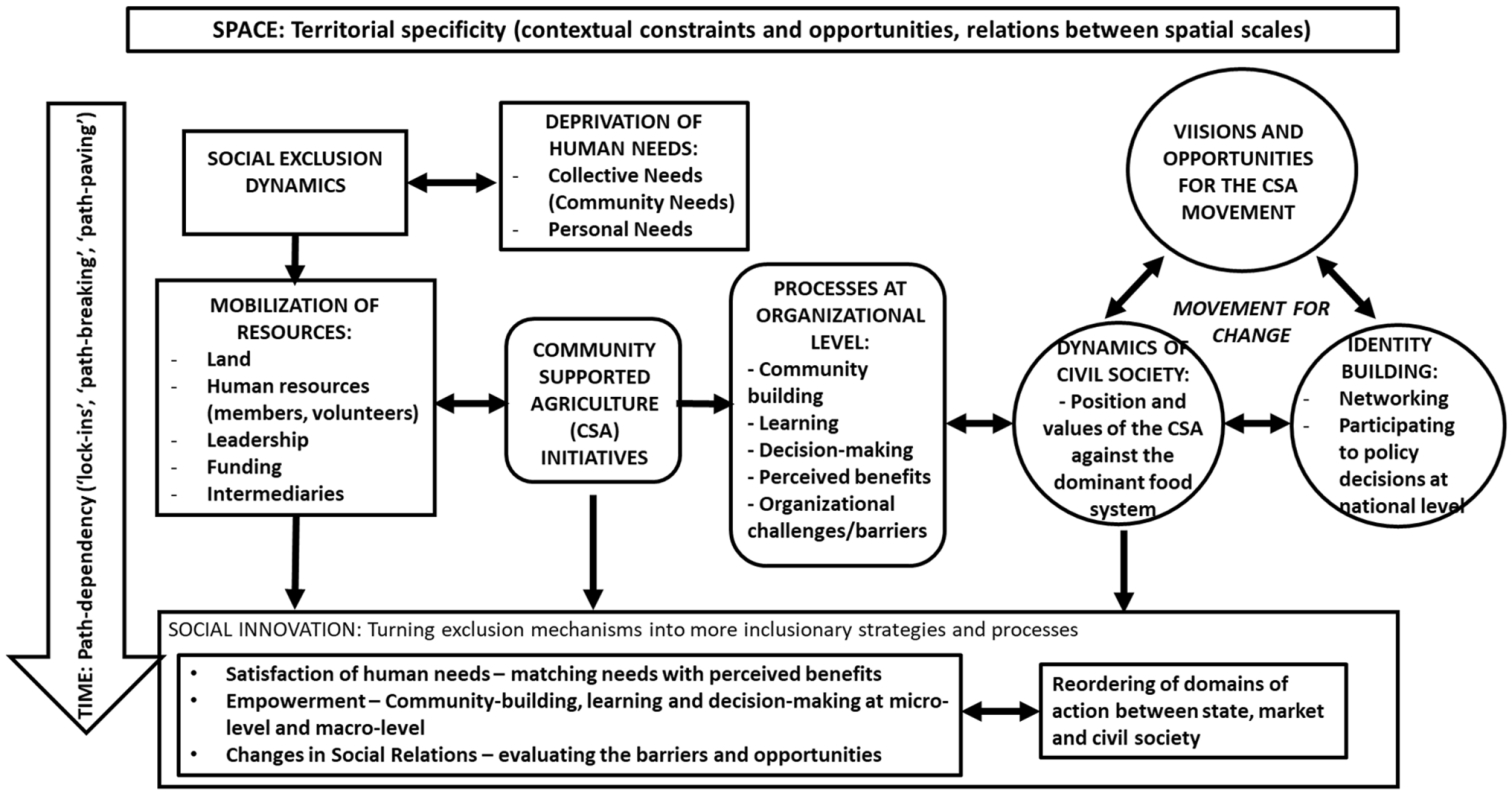
ALMOLIN—themes of analysis for the CSA initiatives. Adapted from González et al. (2010, p. 52)

We based our thematic analysis on ALMOLIN—alternative model for local innovations—an innovative tool that enables mapping the relationship between causes of deprivation of human needs and the way resources are mobilised to create social economy initiatives and a bigger movement for change (Moulaert et al. 2005; González et al. 2010). We utilized the main concepts from the model and added or specified some processes and inputs that were key for evaluating the CSA initiatives in terms of social innovation dynamics, as shown on Fig. 1: (1) ‘Needs’—we specified these as collective and personal; (2) In ‘Mobilization of resources’ we specified the resources used in founding the CSAs; (3) we added ‘Processes at organizational level’; (4) we specified the ‘Dynamics of the Civil Society’; (5) In ‘Identity Building’ we specified two key processes. The names of both the CSA projects and the interviewees were changed for confidentiality and anonymity purposes, in accordance with the research ethics procedures.

## Results

### How the CSA initiatives emerge

In this section we introduce the cases and provide a detailed picture of how resources such as leadership, human capital, land, revenue/funding, and technical support have been mobilized, thereby demonstrating how social innovations emerged and how different CSA models followed different strategies to acquire resources. Summary of the main characteristics of the cases is presented in Table 1 at the end of the section.

**Table 1:** Main characteristics of the CSA cases

Cases	BONT	TYDDEWI	CLWYD	OFFA
Model	Social enterprise with shareholders	Producer-community partnership	Community-led enterprise	Producer-led, owned by a couple of growers
Location	Southeast Wales (10 miles away from a big city)	Southwest Wales (rural farm 5 miles away from a town)	North Wales (3 different sites, close to 2 towns)	Mid-Wales (rural farm between two towns)
Starting Year	2010	2010	2011	2008
Sales	Box scheme (delivery) and farmers’ market	Weekly boxes for the members (picked from the farm)	No sales; food shared weekly among members	Farmer’s markets and shop
Land	5 acres rented	2 acres (of total 70) allocated for the CSA; land owned by the producer	3 acres + 600 m2 from private landlords + 4 polytunnels and an orchard from the local university (free use)	6 acres (4 acres rented + 2 acres owned)
Leaders	Board of 4 directors	Farm owner + core group from community	Chairperson, board of directors, site managers	Couple of growers
Paid staff	Main grower and assistant grower (both part-time)	Grower (part-time)	None	Worker (part-time)
Members	About 100 shareholders	40 members	About 20 members	About 20 members
Volunteers	Regular volunteering days	Regular volunteering days for members + hosting international volunteers	All members involved	Regular volunteering group from the local community
Revenue	Veg box subscription, sales at farmers’ market + some restaurants	Annual membership fee + sales of salad to cafees	Symbolic amount of annual membership fee	Sales at two farmers’ markets and a shop
Financial support	Grants for knowledge transfer, rent (land), growers’ wages, and basic infrastructure	Grants for start-up, grower’s wage, polytunnels, seeds, and a caravan	Grants for start-up and mentoring	Grant for organic conversion

### Bont Market Garden (Bont MG)

Situated on a 5-acre’ land in Southeast Wales about 10 miles away from a big urban centre, Bont MG is a social enterprise registered as industrial provident society for community benefit and has about 100 shareholders—members of the enterprise. The actual growing on the site started in 2010. The garden operates as a box scheme with weekly deliveries to subscribers. Member-shareholders are not necessarily subscribers and for those who are, there is a small discount. Bont MG also sells its produce at a farmers’ market in the nearby urban centre and delivers to local restaurants. The land is rented from a neighbouring community running a forest garden project and hosting events. Part of the initial financial capital of the garden comes from selling one-off shares of £50 each, which can be withdrawn if the shareholder does not wish to continue the membership. There is no monthly or annual subscription fee. Reportedly, more than 100 investors contributed with almost £10.000 in total. This enabled the purchase of a second-hand tractor and building two polytunnels. The enterprise received funding from the Welsh Government via the Rural Development Plan (RDP) under the EU Common Agriculture Policy (CAP), which was used for ‘knowledge transfer’ and another grant from an independent foundation to rent the land, employ a grower-horticulturalist and purchase some basic infrastructure. The project’s revenue comes from sales at the farmers’ market and the weekly box deliveries/restaurant deliveries. However, in order to keep producing, it needs more investment for better machinery, two more polytunnels, and a cold storage. Bont MG is managed by a board of four directors, who brought a range of expertise and skills to the scheme. One of the directors has experience in establishing sustainable businesses and social enterprises. Another provides networking and marketing support, but also has horticultural skills. A third director is a popular figure in the city and has access to a wide range of community groups. And the fourth person is the founding director who is a sustainable food mentor and is directly involved in the garden’s daily issues. Additionally, he is actively involved in the local food council and with other community projects. The directors used their networks and connections to find shareholders and customers, and secure grants from various organisations. The tasks of growing food and organising the weekly boxes is predominantly done by a paid grower and assistant grower, both working on a part-time basis but usually putting more hours than what they are paid for. There are regular volunteering days but very few people come. Reportedly, the engagement level is very low at the annual general meeting (AGM), too. According to the management, those who became members-shareholders were driven by ethical motives rather than desire to invest.

People … have not done the investment for a return; it’s more eco-social investment. In other words, they believe in the values, the ethics and the goal that we have. (Terry, founding director)

### Tyddewi Community Organic Farm (TYCOF)

Located in Pembrokeshire, Southwest Wales, Tyddewi (TYCOF) was initially a private dairy and potato farm since 1938, owned by the father of the current owner and converted in 1996–1997 into organic farm. Inspired by one of the leading CSA farms in the UK, the owner established the CSA scheme in 2010 by allocating land of 2-acres out of total 70. Despite running on a privately-owned farm, the initiative has a distinctive model of producer-community collaboration in terms of management. The CSA produces vegetables and fruits for 40 members and delivers salad bags to some local cafes. This is the only CSA among the four cases that has revenue from annual membership fee, paid on a monthly basis either for a whole share or half share, depending on the membership. Although TYCOF had the advantage of being an established farm with its own land and some equipment, it still needed tools and additional equipment, polytunnels and seeds because it was not a horticultural farm in the past. In addition, it needed to promote the community scheme to attract more members. Establishing a CSA project only became available with a dedicated leadership, particularly from the farm owner who is passionate about food sustainability, and the networking and support of many people and organisations. For example, the local Eco City Group provided a start-up fund and grants for a grower’s salary, a caravan for the international volunteers, and technical support. Other grants came from the National Parks UK and a local CAP group for establishing polytunnels. A bank sponsored seeds as a start off for the first 2 years and the Federation provided technical support. The human capital consists of a paid grower, members, and volunteers. The son of the farm owner is recruited as a grower and paid for 20 h/week, but he works more than 40 h/week. At the start-up stage, the founding members paid for the initial fund, first without receiving any vegetables, and put a lot of time and effort in the project.

We signed up to start paying £30 a month towards [the CSA] as if we were getting vegetables. … We had weeding sessions every week, we had weekly directors’ meetings at that point. It was quite intense as time and energy we put into and lot of enthusiasm as well. (Paul, founding member)

Members can be involved at various levels; there is a voluntary core group, formed of elected members and at least one grower (either the farm owner or his son), responsible for the day-to-day running of the farm. Members can also take part in other groups, e.g. growing, distribution, membership, events or governance and finance. Although volunteering is not essential for the membership, there are regular volunteering days for all members. But most of the help comes from the international volunteers, who come through two organisations: WWOOF UK^4^ and UNA Exchange.^5^ They stay in fully equipped caravans and yurt and are paid weekly for food. At the time of the fieldwork, there were six WWOOF volunteers and about ten UNA volunteers. The growers acknowledge their work as essential for the farm’s success.

If we did not have WWOOF-ers to help us grow and maintain the vegetables, and have the quality we need for our members, we could not succeed. (Roger, farm owner)

### Clwyd enterprise

Clwyd (pronounced Clue-ed) is a community-run social enterprise in North Wales, producing fruits and vegetables for its 20 members. Differently from the other CSA cases, it has three growing places spread over the county with a total area of about 4 acres. Started first as a community garden, Clwyd established the CSA project in 2011 and ran both schemes in a parallel way. However, in 2014 the CSA scheme experienced difficulties and the Clwyd community decided to put it on hold. The CSA model is based on a symbolic annual membership fee and a separate fee for opening a veg account, which then can be credited either with money or time, by doing any kind of work for the CSA. Therefore, some people who spend time growing veg may not have to add money; and the system is based on trust. One of the sites is part of a wooded hillside in proximity to a village. It is a private property, but it was given by the landlord to the use of the Clwyd community for free with a five-year’ agreement. The area was covered in bracken and members had to clear it and prepare it for growing. The second site belongs to the local university and is used for training purposes but four polytunnels and an orchard were allocated with an agreement to the Clwyd enterprise. Situated between the other two sites, it is also used as a hub, and food from all three sites is weighed and distributed here to the members. The third is part of a farm estate that belongs to a local landlord but was given to Clwyd for growing purposes. The enterprise did not use much financial support from other organisations.

We’ve had bits and pieces, but we never managed to get a big chunk. They have tried a few times but not successfully. (Debbie, Chairperson and member)

They used a grant of initial £7.000 from the Welsh Government via the county’s Rural Development Agency for starting up the CSA with a further extension of £3.000 for mentoring. The only income of the enterprise is the symbolic annual membership fee, for the model is not based on an upfront payment from the members. Leadership played a key role in setting up the CSA; the idea and mentoring came from its initial founder who is a professional horticulturalist. Additionally, the CSA had various support from intermediary organisations: The Federation provided ‘moral’ help and has facilitated their networking; Organic Centre Wales, the local Città Slow, Keep Wales Tidy, and the town council provided technical support. Human capital of Clwyd CSA is formed only of its members. From overall 30 community garden members, 20 were in the CSA. Therefore, although the two models were running in parallel, there was overlapping between the members, a group of highly skilled people who provided all necessary support to the CSA, including the financial, marketing and legal advice. At the time of the fieldwork, the paid grower of the CSA had resigned shortly before to start her own enterprise, and many of the members moved with her due to more convenient location. This left the CSA initiative with very few members. The people who remained were trying to re-organise themselves, continuing with the community garden.

### Offa Market Garden (Offa MG)

Producer-led organic garden on a 6 acre’ land, Offa MG is situated between two towns in Mid-Wales. It is run by a couple of growers who first established the garden in 2008 on 4 acres of rented meadow with a shed, where they lived in a caravan with their children. Three years later, they bought 2 more acres of land to expand the initiative to 6 acres with three polytunnels, a greenhouse, a purpose-built packing shed, and an eco-house for their family. Half of the investment was financed personally by the family, and the rest was paid with small grants and interest-free loans from the landlord and a trust that lends to small-scale organic growers. Certified organic since 2010, Offa MG received a grant from the Welsh Government through the Organic Centre Wales and technical support as part of a package for conversion to organic growing. Apart from a small grant from the local council, the initiative did not use other financial support. The garden sells its fresh produce at the farmers’ markets in the nearby towns and to local pubs and restaurants. In 2015, Offa MG also started selling its produce at a local shop opened jointly with another retailer. The CSA scheme is based on a voucher system rather than membership fee. Community members receive free introductory box of fruit and veg when joining the CSA and then buy voucher books of £200 to purchase their food from the farmers’ markets or the shop. The overall number of the CSA members is about 20. Part of the members only support the grower financially by buying vouchers and do not help on the field. The other part is a group of 6 to 12 people who call themselves the ‘Weeding Group’ coming on a regular weekly basis to help with any growing tasks. One of the volunteers was offered later a part-time job for 1 day a week or 2–3 days in the high season. Separately from the ‘Weeding Group’, there are volunteers who help with organising the volunteering sessions or promoting the initiative. Volunteers are predominantly retired people.

We are coming from the older end of the spectrum and quite many of us are grannies …, because we have the time. Maybe financially we are a bit more solvent.” (Lynn, volunteer)

But there are also younger people in the group who bring their children, and that is why the ‘Weeding Group’s sessions are usually organised later in the evening, after the workday or school. The grower explained that it was very easy for him to find volunteers to help, which demonstrates the existence of a supportive community in the area, willing to give their time and work for the cause.

### How the CSA initiatives satisfy unmet needs

To address the first gap related to the product dimension about identifying the needs, we explored the *collective needs* that started the initiative, and the *personal needs* for people’s involvement. Then we identified the *perceived benefits* that people get from these initiatives and compared all three for each case to address the second gap and find out if the needs have been met. We summarized the results in Table 2. According to the results, the most important need that triggered the emergence of the initiatives and people’s involvement was the need for good quality local and organic vegetables, which is similar to the findings in studies about the CSA motives (Cox et al. 2008; Sharp et al. 2002; Farmer et al. 2014). The exclusionary circumstances that this need created was articulated as deficit in terms of vegetable production in Wales, or lack of local and organic produce in the area. However, behind the need for local and organic food, there were more complex drives, such as wider concern for sustainability and the environment.

We weren’t really driven by a desire to make a business. … We were interested in being part of contributing to not only a sustainable food chain for Wales, but also a lower carbon Wales. (Terry, Bont MG).

For the producers, it was more important to reconnect people with the source of their food by eliminating the food miles and packaging, and by respecting food as something valuable rather than commodity:

I liked the whole principle of it: feeding local people direct from the field and having people care about the farm and about the source where food came from. (Roger, TYCOF)

Members had broader ethical concerns behind the need for local and organic food, too. But in addition, they also wanted to be part of a community and to gather, talk, and share experiences with like-minded people, while at the same time provide livelihood for the grower.

It fits my values and I have always been fascinated by food and the idea of coming together with people to grow substantial amount of food; but also, to provide the livelihood of [the grower’s] family was a good idea. (Paul, TYCOF)

Other, less articulated need for the founders was to create employment and educate people who wanted to establish their own small-scale organic food growing projects, while for members, learning with the aim to set up their own business and spending time outdoors, were other personal needs.

**Table 2:** Matching needs with benefits

	Community needs	Personal needs	Personal benefits
BONT	Supply of organic vegetables		Fresh organic food
	Transferring knowledge and skills about growing food	Learning with the aim to set up own horticultural business	Learning
	Creating employment	Doing something different from their usual job/ gardening as a hobby	Earning modest wage (grower)
	Contributing to the environmental sustainability	Attracted to the ethical aspects of the project	Supporting organic food growing
		Meeting other people	Social contacts
		Having free time due to retirement or unemployment/desire to help	Fresh air and being outdoors/staying fit
TYDDEWI	Need for local and organic produce in the area	Need for local and organic vegetables	Good food, vegetable share
	Having people care about the farm	Growing food together in a community	Sense of community, social environment
		Provide livelihood for the farmer	Contributing towards sustainability
	Reconnecting people to the source of their food without the	Learning about sustainable, organic agriculture	Learning, sense of achievement
		Working outdoors in a farm	Therapeutic benefits, being in a beautiful environment, access to a real farm
			Accommodation (volunteers)
CLWYD	Need for local and organic vegetables in the area	Need for local and organic vegetables	Good quality, fresh vegetables/cheap, properly grown, nice food
	Growing food in a community	Socialising, being in the community	Being in a community/teamwork
		Learning about growing food/growing own food	Learning
			Physical and mental health
OFFA	Need for a good quality local and organic food producer in the area	Good quality, organic food	Good quality, fresh organic food
		Supporting the values of growing local and organic food, helping a good cause	Sense of fulfilment of doing something positive and productive
			Sense of achievement/s eeing the results of the labour
		Social side, growing food as part of the community	Meeting others in the community
			Nice place to spend time
			Physical and psychological wellbeing/fresh air and physical activity

Comparing the needs with benefits clearly demonstrates that each CSA initiative is a medium for satisfying the needs that caused deprivation and triggered the social innovation. Firstly, local and organic fresh food was both community and personal need. And respectively, food is the main benefit that people get from their involvement, which participants describe as ‘fresh’, ‘organic’, ‘good quality’, ‘cheap’, ‘nice’, ‘decent’, and ‘grown properly’. The need for ‘growing food together in a community’, ‘socialising’, or ‘meeting other people’ was also reported as a benefit. For some participants, it was even more important than getting quality food. Ethical concerns expressed as needs for ‘supporting local farmer’, ‘supporting the values of growing local and organic food’, and ‘supporting a good cause’, were reported as benefits of ‘supporting organic food growing’, ‘contributing toward sustainability’, and ‘sense of fulfilment of doing something positive’. Meanwhile, although mental and physical health was stated neither as a community need nor as a personal motive, it was among the benefits in all cases, which demonstrates that the CSA initiatives provide additional benefits beyond satisfying unmet needs. Participants described nature as ‘beautiful’, ‘relaxing’ and ‘real environment’, and stated that it helps them connect to themselves and contributes to their mental health. Some spoke about the ‘medical benefits of having your hands in the soil’. Others emphasised the physical health benefits of working in fresh air and outdoors. In sum, results show that the Welsh CSA initiatives provide the means of satisfying the needs that created social exclusion and triggered their emergence.

### How the CSA initiatives empower people

Related to the empowerment dimension, we explained earlier that the issues about social capital are well documented in the literature but there is no evidence about other ways of empowerment. Therefore, in addition to the sense of strong community, we examined learning and decision-making as two processes in the CSA initiatives that can enhance the capabilities and the voice rights of people. Building strong community and social capital in the CSA initiatives is one way of empowering the community. Our results show that the model of the CSA has an impact on the type and quality of the community. The strong sense of community was more tangible in two of the cases where the grower was the leader of the initiative, namely TYCOF and Offa MG, because the growers play bonding role for the members and volunteers. From the growers’ point of view, there is a mutual recognition of the significance of each side to the other side. At TYCOF the community organizes events and gatherings separately from the volunteering times; they have feasts together when a member bakes pizza for everybody in a clay oven. Also, the weekly vegetable boxes are not delivered to the members but picked from the farm. However, some members argued that the community was not as strong as the farm owner would have liked due to many people not being interested. Low level of engagement was a problem in Bont MG, too. The work was mainly done by paid growers who came on part-time basis. The founding director was uncertain about the strong sense of community due to the low level of engagement both on the field and at the AGMs. The community-led case, Clwyd, had different problems related to social capital. One of the challenges was the distance between the three growing sites, which was partly overcome by designating one of the sites as a hub for food sharing. However, the biggest challenge was to introduce the CSA scheme separately from the existing community garden.

Those people who had come from community garden roots … were very committed to giving what was asked of them when they moved to the CSA. The people who did not come from that background … took advantage and they did not put that hours in. (Kelly, founder of Clwyd CSA)

After losing considerable part of their members, who left with the resigned paid grower to join her enterprise, only a small group of dedicated and loyal members remained in Clwyd, thus showing the real cohesive group.

Learning as another way of empowerment increases the capacity of individuals and communities to produce their own food, acquire essential life skills, connect with like-minded people, and learn about sustainability issues. Therefore, learning is not only a benefit but a vital process in maintaining the initiatives. Very few people in all four cases had formal horticultural training. The members and volunteers usually learn while working on the garden/farm. Practical food growing is the main skill that they learn. However, it can include some advance knowledge, e.g. different pests and diseases, seasonal changes and organic principles, such as crop rotation, companion planting, and soil care. There is no formal process, and people learn by watching the grower or horticulturalist, then doing it on their own. At Clwyd, the horticulturalist gives members agency by making them teach other members how to do certain things, which helps people memorise well all the process. Additionally, transfer of skills can make people economically powerful by acquiring a job or setting up their own food growing enterprises. Meanwhile, teaching gardening skills is not the main aim for some of the initiatives. For example, at Bont MG the leaders were interested in teaching horticulture at entrepreneurial level, and the garden provided courses on the field to apprentices who wanted to become horticulturalists, through a government-funded training and employment project, Horticulture Wales, which was planned to come to an end in August 2015.

People learn about some of the difficulties both of trying to grow on a small scale like this and growing wide range of crops, but also difficulties in dealing with customers. (Ryan, paid grower at Bont MG)

Learning also involves communication skills, or ‘people skills’ and multicultural skills due to meeting different people, specifically volunteers from all around the world in case of TYCOF. At the same time, the practical involvement in producing food teaches people to appreciate the hard work that it takes, as often emphasized by members and volunteers. But more importantly, they also learn about sustainability and environmental issues, and the importance of the CSA, often discussed in informal conversations. This is also referred to as ‘second-order learning’ (Seyfang and Haxeltine 2012; Marsden 2013). Table 3 summarizes the learning across the cases.

**Table 3:** Learning in the CSA

Learning	BONT	TYDDEWI	CLWYD	OFFA
Basic gardening/horticultural skills	✔	✔	✔	✔
Advanced horticultural skills (organic gardening growing)	✔	✔	✔	✔
Communication skills	✔		✔	✔
Sustainability Issues	✔		✔	
Managerial skills/planning/dealing with customers	✔			✔
Variety of vegetables		✔	✔	
Running small horticultural business	✔			
Multicultural skills		✔		

Decision-making is another way of empowerment, as it enhances people’s ‘voice rights’ (Martinelli 2010, p. 42). The ability of people to have their say about the processes or management of the CSA initiatives is empowerment at micro level. In two of the cases—TYCOF and Clwyd—members are given the opportunity to take part in decisions at all levels by being involved in core groups or board of directors. Any willing member can take part in these groups, which is a way of including everyone and ‘eases the burden’ of the grower.

Everybody is fully involved in the decision-making. … People want to have ideas; they are actually inviting me to give them new ideas. (Ruth, Clwyd)

At Bont MG, daily decisions are left to the growers. At a higher level, these are taken by the directors. Members can participate to the AGM, where they can bring important issues and vote for decisions. And at Offa MG, the decisions at all levels are taken by the growers. But whatever the mechanisms, members and volunteers in all cases feel that their suggestions are taken into consideration, formally or informally, which is a form of democratic governance at micro-level, also described by Defourny and Nyssens (2013) as a recent trend of diversification of the actors in social enterprises working on the same project, where even users and suppliers work and manage together. Comparative summary about decision-making is provided in Table 4.

**Table 4:** Decision-making in the CSA

	Daily decisions		Higher level/managerial decisions	
	Who takes the decisions	Members’ participation	Who takes the decisions	Members’ participation
BONT	Main grower	Informal contribution	Board of Directors	Voting participants at AGM
TYDDEWI	Main grower + growing group	Informal but can take part in the growing group	Core group	Active participation
CLWYD	Core group + site meetings	Active participation	Directors (elected members)	Voting participants at AGM
OFFA	Growers	Informal contribution	Growers	n/a

### The potential of the CSA in Wales for social change

Regarding the process dimension, to address the first gap, we explored how the people in the Welsh initiatives see the CSA and its values compared to the dominant food system. Results demonstrate that participants think of the CSA as a very small part of the main food system, described as ‘relatively minority’, ‘fringe’, ‘tiny part’, ‘marginal’ and ‘so small that does not have any impact at all’. One reason is the fact that the CSA is still very limited in numbers—nearly 100 in the UK, of which ten are in Wales. And the second reason is that few people know about the CSA as most people are either unaware or do not care about sustainable food; those who care were described as having “in their souls the idea of growing food, community growing and sharing things” (Lynn, Offa). The CSA is also viewed as complementary to the main food economy by providing inclusive environment for different parts of the society, e.g. people with disabilities or health problems, or low-income families. And despite currently being ‘tiny’ part of the main economy, people believe it will become bigger because the movement ‘is building up knowledge and skills that could be expanded’ (Dave, Offa) and ‘that is how change happens’ (Terry, Bont). When asked to compare the values of the CSA to those of the dominant food system, all interviewees expressed unequivocally that the two have different values. In sum, they explained the values of the CSA as organically, locally and sustainably produced food; polyculture on a small scale; balancing profitability with environmental sensitivity; bringing value to the food; and sharing and cooperation. These were juxtaposed to the conventions of the dominant food system, expressed as intensive farming by using harmful chemicals; profit at any cost by creating externalities; cheap food; and competition rather than cooperation.

To address the second gap related to the process dimension, we explored the difficulties and opportunities for scaling up for the different CSA models, and their visions for movement. We summarized the results in Table 5 at the end of the section. In terms of difficulties at organizational level, *insufficient human capital*, or not having enough members or volunteers was the primarily reported challenge. The reasons behind the need for more members varies between the different models. Bont MG needed more shareholders to finance new equipment and more polytunnels to grow more quantities and speed up the work, while TYCOF needed at least 65–70 members in total (currently has 40) to be able to both provide livelihood for the farmer and feed its members. And Clwyd needed more members to-re-launch the CSA scheme that had been put on hold. Second major difficulty was related to *accessibility*. For instance, Bont MG is situated about 10 miles away from a big city and a few miles away from a town, but this creates a ‘psychological barrier’ of remoteness. In case of Clwyd, reaching the CSA site even from the nearby village is difficult. Also, none of the CSA initiatives are on a main public transport route. *Lack of initial capital* was another big challenge that can be even worse for the communities not possessing land, which is why some participants spoke of the need for support package from the Government at the start-up phase. *Inadequate equipment*, e.g. machinery and cold storage, *marketing difficulties*, and *time-constraint* were other challenges. All these organizational difficulties create lock-in at micro-level in reaching economic viability. As for the macro-level barriers that relate to the CSA movement, firstly, the place of the CSA in the Welsh food and agriculture policies is almost non-existent. For example, policy documents like the Farming Strategy for Wales (WAG 2009) or the Food Strategy (WAG 2010) do not include any arrangements to promote and support the CSA in Wales. Secondly, the mechanisms that enable growers and members of the CSA initiatives to directly take part in policy-formulations, which is also empowerment at macro-level, are limited. There were very few occasions when such participation happened, e.g. taking part in the consultations of the Welsh government about reshaping the organic agriculture framework within the EU CAP. Third barrier for the movement is the *inadequate formal horticultural education* in the country and lack of programmes in colleges about growing food. And the final reported macro-level barrier is *low financial reward* for horticultural producers that makes the profession unattractive especially for young people.

**Table 5:** Process dimension of the CSA

	BONT	TYDDEWI	CLWYD	OFFA
	Shareholding social enterprise	Community–producer partnership	Community-led social enterprise	Producer-led enterprise
Organizational (micro-level) barriers	Location is difficult to reach on public transport	Insufficient number of members to provide livelihood for the grower	Remote location that is difficult to access	Marketing the voucher scheme and the idea of CSA to more customers
	Insufficient equipment (second-hand tractor)	Not enough people working on the farm	Not enough number of dedicated members	Time-consuming, hard work
	Insufficient facilities, e.g. cold storage	Lack of demand for the produce from the local shops and restaurants		Pests
Current status/needs	Depends on funding to pay grower's wage	Has 40 members and provides half of the grower's wage	CSA scheme on hold; growing continues as a community gardening at 3 different sites	Self-sustainable and owns part of the land but growers work long hours
	Needs more shareholders to provide sources for two more polytunnels, a new tractor and cold storage	Needs at least 65–70 members/families to be self- sustainable	Needs a new place close to the town and new members to re-launch the CSA	With the help of 20 members it provides livelihood to the growers’ family
Vision/aims (organizational level)	To be self-sustainable	To be self-sustainable with a bigger community (more members)	To re-launch the CSA with more members on a more acessible land	To keep the initiative sustainable and provide livelihood for the growers’ family
	To be a model for economically viable small- scale horticultural enterprise and inspire others to increase food production in Wales	To improve facilities, e.g. temperature-controlled storage	To do more marketing and promote the way of eating locally and sustainably	To get more efficient by refining the growing methods and to supply the nearest towns with locally grown, organic vegetables
Barriers on macro-level	Lack of governmental policy (strategy or action plan) regulating or aiming at promoting and developing community food growing in Wales	(1) Lack of support payments from the Government suited to small producers; (2) lack of promotion of the CSA by the government; (3) not recognized by farmer unions	(1) Lack of support of the idea of the CSA; (2) insufficient formal horticultural training	(1) Low financial reward for horticultural producers; (2) insufficient formal horticultural training
Networking	Member of the CSA UK Network, FCFCG, Soil Association and Organic Centre Wales	Member of the CSA UK Network, the Welsh CSA group, FCFCG, Soil Association	Member of the CSA UK Network and FCFCG	Member of the CSA UK Network, FCFCG, the Soil Association and Organic Centre Wales
	Taking part in their events	Hosted annual gatherings	Hosted annual gathering and had visits to/from other gardens/ farms through events	Involvement with organic growers’ alliance
	Had visits to/ from other community gardens/farms	Minimal collaboration with a local Transition Town initiative	Connections with the local Citta Slow movement and other community groups	Involvement with other local community projects by giving talks
Taking part in decision-making at macro-level	Indirectly, via membership at the city food council	Taking active part in the international gatherings of the CSA network	Taking part in a scoping study about potential places for setting up new CSAs in Wales (funded via the FCFCG)	Taking part in meetings about reshaping the organic framework within CAP and in talks of Welsh organic growers with the minister for agriculture
Visions for the movement	CSA is one of the many solutions for transitioning towards more sustainable food systems	CSA has a huge potential to become exponential in numbers and provide jobs for young people who want to get into farming and growing	If more farms get involved, the CSA model can help the struggling farmers because the community can share the risks	Rather than purely community-led food growing, a combination of business and community might result in more successful initiatives
	Need for (1) more coordinated governmental policy toward facilitating people’s access to land and (2) support from the communities either by volunteering or buying the produce	Need for (1) promoting by the Government the idea of the CSA as a sustainable way of producing food and (2) support package for establishing CSA projects with financial aid for land and grower's wage	Need for (1) cheap land by the Government and councils for communities to establish CSA with financial support, loans, and equipment- sharing (similar to the AMAPs in France) and (2) more formal education programs about growing food	Need for (1) better financial reward for the horticultural producers and better value for the food produce, and (2) more formal horticultural training with programs about growing food

In addition to the barriers at micro and macro levels, we examined the networking as an opportunity for scaling up. The launch of the CSA Network UK in 2013 and the Tyfu Pobl (Growing People) program by the Federation have facilitated the networking between the initiatives, which can be interpreted as change in governance relations at meso-level. One way of networking is by taking part in the regular national or regional gatherings organised by these organizations. Two of the cases, TYCOF and Clwyd hosted annual CSA gatherings in the past. Networking also happened by exchanging visits with other CSA farms and gardens to share knowledge and skills, for which the Federation provided travel bursaries.

It was good to see a much bigger CSA and how they structured themselves … it was a very useful visit. (Will, TYCOF)

Meanwhile, some participants suggested that attracting more farmers to the CSA schemes through farmer unions is another way of scaling up the movement that can help the struggling farms and create employment.

The community can help the farmer to survive because not many small-scale farmers are making a good living, they are struggling … [They] can get together with enthusiastic communities locally to do it. Then that may be the way forward, which was the original concept of the CSA model. (Trevor, Clwyd)

The final gap in the literature related to the transformative potential of the CSA initiatives was the lack of concrete examples of how they address social injustice and exclusion. The Welsh cases have different mechanisms for making the schemes accessible to everybody. In Clwyd people can participate without paying and can take food in exchange for their work for the initiative, i.e. pay with their time. TYCOF keeps the membership fees at minimum level and allows people to pay ‘as much as they can afford’,^6^ which is openly stated on their website. Although the other two initiatives do not have direct ways to make their membership affordable for everyone, they always accept volunteers and offer them fresh produce in exchange for their help. Regarding the participants’ visions for the CSA movement, our research highlights that CSA is considered ‘definitely’ part of the solution for transitioning towards sustainable food systems with the potential to contribute “enormously” to the food sustainability by boosting the local economy, creating jobs and reconnecting people to the source of their food.

## Discussion

Related to the product dimension, although the Welsh CSA initiatives have different models and characteristics, they all emerged as social innovations in reaction to similar needs. Secondly, according to ALMOLIN model (González et al. 2010), unmet needs cause deprivation and social exclusion, but in our CSA cases there is no food deprivation per se; the primary need is specifically for locally and sustainably grown, ecologically sound and possibly organic food for people who have broader concerns for the food systems and the environment. All other needs, e.g. being part of a community, supporting sustainable enterprises and learning, are clustered around the food growing as practice. And a third point is that many of the needs are currently not urgent but can become urgent as participants often spoke about the probability of a fuel crisis leading to food scarcity, which is interesting because crisis is considered the second drive for social innovation after needs, and the two are interrelated (Baker and Mehmood 2013). Moreover, the fact that participants in the CSA schemes are people with concern for the environment and sustainability supports Seyfang’s argument (2009, pp. 72–74) that ideology can be another driver for social innovation. Therefore, the Welsh CSA community can target people who support environmental sustainability and value the sharing and community aspects, also suggested by Goland (2002). Future research may examine if the antecedent needs change over time and whether these differ in places where ecologically grown, local and organic food is widely available.

Regarding the empowerment dimension, learning as an empowering process increases the capacity of people to produce their own food thus making them more resilient. Moreover, learning equips individuals with skills that makes them economically powerful by acquiring jobs or starting their own enterprise. One example is the main grower of Bont MG, who had joined as a volunteer but later offered the job and currently has his own horticultural organic enterprise. Another example is the assistant grower of the same initiative who later acquired a job for setting up a new CSA. In a similar way, a founding member of Tyddewi started a new CSA initiative. These are all examples of how learning can lead to transfer of skills that also strengthen the movement by replicating the innovation. In terms of participation in decision-making, the results demonstrated that CSA cases are places for empowerment at micro level. However, when it comes to decision-making at macro level, participants do not feel empowered enough due to two reasons. The first is the lack of national policies or strategies for promoting community food growing, and the second reason is the limited possibility for the CSA communities to participate in the food-related policy decisions at national level. Occasions when leaders of the CSA are invited to discuss policies are extremely rare, and response from community members to government consultations is usually low due to lack of information, lack of time or because these are considered as ‘closed-doors’. This brings forward two questions. Firstly, what are the factors that limit the participation of the CSA initiatives in consultations and policy decisions at national level? And secondly, how can this situation improve and what mechanisms can be developed to enable it? To sum up, the CSA in Wales empowers individuals and communities at micro level, within the organization, but the movement must get stronger in order to be empowered at macro level as well. As Miquel et al. (2013) explain, if citizens’ political capacity is strong, they can influence institutions in their policy decisions; but if they are not mobilized enough, their influence remain within the boundaries of their community.

Related to the process dimension, we discussed earlier that AFNs have been criticised for their failure both to be more socially just (Goodman 2004; Guthman 2008a; Farmer et al. 2014) and to oppose the neoliberal food regime. We already examined the different ways the Welsh CSA schemes developed to be more socially equitable, such as alternative payment possibilities. Another suggested way for the CSA schemes in addressing social injustice is by playing active role in food deserts, i.e. areas with limited access to fruits and vegetables (Mader and Busse 2011). To do that, the Welsh CSA initiatives first need to overcome the lock-in about acquiring land that is close to cities and towns and accessible to more communities. Land as a resource is considered the biggest challenge for the communities (Armstrong 2000; Henderson and Hartsfield 2009). The Welsh communities used great creativity in gaining access to land by collaborating with local landlords in various forms of agreement, also discussed by Franklin and Morgan (2014). However, they think that the Government and councils should make available land for food growing communities at more accessible locations. And although the Welsh Government awarded funding in 2018 to Community Land Advisory Service (CLAS) to help at least 50 communities every year to access and own land (CLAS 2018), it barely made difference for the CSA. As for the second criticism about the failure of the food movements to oppose the neo-liberal regime, establishing the position of the CSA against the main food economy and comparing the values of the two systems helped us determine the CSA in Wales as a ‘strong alternative’ for two reasons. Firstly, although the CSA is very small in its position against the corporate food economy, it is not subordinated by the latter (Watts et al. 2005); the initiatives can rather be defined as autonomous food spaces, separate from the corporate food system, as suggested by Wilson (2013). And secondly, the CSA initiatives use the conventions of the moral economy as opposed to the market economy (Follett 2009), e.g. human and animal welfare, community-building, ecological sustainability, and trust and transparency in relations. All these values are also in line with the principles of food sovereignty, which is considered as having the best potential to make real transformation of the food systems through its political stance of rejecting the neoliberal food governance (Fairbairn 2012) and clear opposition to the trade liberalization of food (Alkon and Mares 2012). Moreover, Welsh CSA Network is part of the International Network for CSA Urgenci, which openly states its involvement with the European movement for Food Sovereignty since 2011 with its focus on countering the expansion of supermarkets via short supply chains and providing food for everybody regardless of their income (URGENCI n.d.).

The major limitation of this study is that the results cannot be generalized despite the big number of cases. In addition, they are predominantly based on perceptions and observations reflecting a relatively short period of time. Nevertheless, this study contributes to the literature by studying the processes and factors that enable and constrain the transfer of social innovations from micro-scale opportunity spaces into macro-scale “workable utopias”. Additional contribution is the use of ALMOLIN as an analytical tool and its adaptation to the CSA cases, which allowed the evaluation of the social innovation initiatives from their emergence to their transition to a bigger movement and impact on social change at various levels. We also identified several questions that might contribute to a future research agenda: One question is whether needs change over time or differ in areas with access to sustainably produced food. Second question is about the factors limiting the participation of the CSA initiatives in consultations and policy decisions at national level. Third question is about the reasons for the slow replication of the Welsh CSAs given the funding and support of intermediary organizations. Another relates to the reasons for non-participation of local communities to the CSA. And the final questions are about the attitudes of Welsh farmers towards the CSA and possible ways for scaling up by establishing collaborations with other types of social innovation.

To have an impact on the food policies at local and national level, negotiate support from the Government and fairer prices for producers and get promoted, the CSA movement in Wales needs to grow through replicating the initiatives and scaling up. Replication of the projects in a horizontal way is metaphorically compared by Deleuze to rhizomes with underground network of roots linked to each other (Scott-Cato and Hillier 2010). It is also defined as ‘expanding de-commodified spaces’, referring to places that challenge the corporate regime (Calvário and Kallis 2017, p. 598). Replication of the CSA is already happening in Wales, although at slow pace. The number of initiatives was expected to grow since a scoping study commissioned by the Federation identified 20 potential places for setting up new projects (Groves 2015). It is worth exploring why these projects have not emerged for five years since the results of the study, especially considering the support with land provided by CLAS. In addition, further research is needed to survey the reasons for non-participation to the CSA by the local communities. On the matter of scaling up, hybrid strategies might be one possible way for the CSA. Although different types of hybridity are suggested for scaling up the CSA, e.g. through commodity practices such as labour, seasonality, and addressing customer expectations (Nost 2014), our argument is about hybridity by involving mainstream actors, which was reiterated by many participants of the CSA cases and also discussed by Seyfang and Haxeltine (2012) as a way of promoting grassroots community initiatives. Further research is needed to establish what is the attitude of the Welsh farmers towards the CSA. Additional question is how the CSA initiatives can preserve their alternative values in the case of involving farms as mainstream actors. Also, an idea worth researching is the possibility of scaling up the CSA by establishing collaborations with other types of social innovations, e.g. community energy and housing, alternative currencies or transition movement. And finally, some studies suggest that alternative food initiatives can become part of urban food strategies (Wiskerke 2009; Matacena 2016), which can have a huge impact on the promotion and transformative power of the CSA in Wales. The question is, how can the Cardiff example as the only city with urban food policy can be replicated in other areas as regional food strategies.

## Conclusion

Reflecting back on our main research question of how the CSA initiatives in Wales can become ‘workable utopias’ for food systems’ change through social inclusion and empowerment, we demonstrated that the Welsh CSA cases analysed here show great variety in their characteristics, processes and possibilities. However, they all play distinctive roles as part of the social economy in satisfying the needs for ecologically sound and ethical food, grown within communities of like-minded people and empowering individuals and communities at micro level. Moreover, the CSA initiatives are places where communities experiment with producing different crops on a small scale and finding ways to become economically sustainable and resilient, thus contributing to gradual transformation by building knowledge and skills and raising awareness, also termed ‘quiet sustainability’ (Kneafsey et al. 2016). The type of the CSA affects the financial sustainability of the initiatives as the results show that the purely producer-led type of CSA is the most self-sufficient among the cases while on the contrary, the community-led model is the most vulnerable. In order to become ‘workable utopias’, the CSA initiatives need to overcome the barriers that prevent them from replicating, participating in policies and decision-making at macro level, and scaling up.

The COVID-19 pandemic is increasingly regarded as an opportunity for transforming the unsustainable and unjust global food systems. Food scholars focus on various features that the new food systems must incorporate. Resilience and the ability to ‘bounce back’ in the event of drastic change is one feature that is repeatedly articulated (Worstell 2020). In addition, the call is for more equitable, healthy and ecologically-sound, decentralized and distributive systems based on democratic governance at all levels, (Moragues-Faus 2020; Blay-Palmer et al. 2020). It is also suggested that the transition must be towards systems found upon the principles of agroecology with solidarity and circular economies and strengthening the local food value chains (Gemmill-Herren 2020). Social capital, cooperation of people and communities, and collective management of resources as well as re-orienting policies to support communities and protect livelihoods is regarded as essential for the way forward (Pretty 2020; Graddy-Lovelace 2020). It seems that community supported agriculture has an important role to play in the future as it embodies all the features considered for more sustainable food systems: it is solidarity-based, equitable, ecologically sound, and healthy. But most importantly, the CSA has demonstrated for now that it is resilient in times of crisis and not only provides food but nurtures communities and cares for the vulnerable people.

## Footnotes

1 ‘Path dependency’ or ‘path dependence’ is the tendency of institutions or technologies to become committed to develop in certain ways as a result of their structural properties or their beliefs and values (Encyclopaedia Britannica). It can create either ‘lock-in’ situation that blocks the change, or ‘path-paving’ when it supports the social innovation, or ‘path-breaking’ when it leads to a sudden transformation (González et al. 2010).

2 The UK’s leading organisation for promoting sustainable food and farming and the largest organic certification body in the country.

3 Finding the cases was facilitated by the participation of the leading author to the CSA Network’s launch conference in December 2013 in Stroud where she made connections with some of the Welsh initiatives.

4 The WWOOF—World Wide Opportunities on Organic Farm—is an organisation that was established in 1971 in the UK but quickly grew and became an international organisation. It brings together organic farms and gardens throughout the world with volunteers who want to do practical work They are not expected to have farming or gardening skills prior to coming to work because they learn it on the site (WWOOF_UK n.d.).

5 UNA Exchange is a Wales-based international volunteering organisation founded in its present form in 1973, but with a history of almost 100 years as a volunteering movement. It works with partners in 70 different countries and provides volunteering opportunities in projects in different areas of work, designed with local organisations (UNA n.d.).

6 This CSA farm wants to put into practice a payment system that has been successfully implemented in Freiburg (Germany) according to a member who attends the European and international CSA meetings. The system allows each member to offer anonymously the amount they can afford to pay, and all the sums are revised until they add up to a level that provides livelihood to the grower. Thus, rather than imposing everybody the same fee, people are given the opportunity for fair distribution of the payment.
